# Global Trend in the Research and Development of Acupuncture Treatment on Parkinson's Disease From 2000 to 2021: A Bibliometric Analysis

**DOI:** 10.3389/fneur.2022.906317

**Published:** 2022-07-08

**Authors:** Xiaoping Li, Wan Wei, Yuan Wang, Qiang Wang, Zhibin Liu

**Affiliations:** ^1^The Third Clinical Medical College of Zhejiang Chinese Medical University, Hangzhou, China; ^2^School of Acupuncture-Moxibustion and Tuina, Shanghai University of Traditional Chinese Medicine, Shanghai, China; ^3^Innovation Research Center of Acupuncture and Medicine, Shaanxi University of Chinese Medicine, Xianyang, China; ^4^Shaanxi Key Laboratory of Acupuncture and Medicine, Xianyang, China

**Keywords:** acupuncture, Parkinson's disease, bibliometric analysis, global trend, Traditional Chinese medicine

## Abstract

**Background:**

Acupuncture has been widely used in the treatment of patients with Parkinson's disease (PD) in the world. Despite we have an in-depth understanding of acupuncture in this field over the past years, there is no available literature on bibliometric analysis on the development of acupuncture on PD. This study was designed to explore the global trend in the research of acupuncture on PD in the recent 20 years by the software CiteSpace (5.8.R3) and VOSviewer (1.6.14).

**Methods:**

Publications regarding acupuncture therapy for PD from 2000 to 2021 were retrieved from the Web of Science Core Collection database. CiteSpace and VOSviewer were used to analyze the number of publications, the contribution of countries, institutions, journals, authors, references, and keywords.

**Results:**

A total of 217 studies were extracted from the database. The outputs of the publications in this field showed an upward trend during the past two decades. The country and institutions with the most publications in this field are China, South Korea, and the USA. They were the main contributors to the research. Kyung Hee University and Capital Medical University were the two most productive organizations. Hi-Joon Park had made the greatest contributions to the field. *Evidence-based Complementary and Alternative Medicine* was the most popular journals in this field. “Electroacupuncture” and “Bee venom acupuncture” were emerging research hotspots.

**Conclusion:**

The research on acupuncture on PD is potential. Authors from different countries/regions and organizations need to remove the language and academic barriers to enhance global cooperation and communications. Scholars in this field need to publish their research findings in high-quality journals to gain more attention worldwide. This study indicated that the mechanism leading to the non-motor symptoms of PD, the establishment of appropriate models that fully reflects the non-motor features of human PD, and the efficacy and safety of promising therapies for patients with PD will remain research frontiers in the future.

## Introduction

Parkinson's disease (PD) is recognized as the second most common neurodegenerative disease with clinical features such as postural instability, resting tremor, bradykinesia, and rigidity (1). These motor symptoms are the main clinical criteria leading to the diagnosis of PD (2). However, patients also suffer from non-motor symptoms that seriously challenge their living quality in the early phase of the disease (3). Therefore, both the motor symptoms and non-motor symptoms are the concerns for patients with PD. The incidence of PD is high and increases rapidly after the age of 50 years (4), which brings a huge economic and medical burden to society (5, 6).

Acupuncture, a traditional treatment with few side effects from China 2,000 years ago, is used for different kinds of diseases worldwide (7, 8). It has been reported that around 40% of patients in the USA take complementary and alternative medicine when they deal with their illness. In South Korea, this can be as high as 76% (9, 10). Acupuncture is an essential complementary component to the drug therapy for PD (11). The mechanisms of the effectiveness of acupuncture for the treatment of PD remain uncertain, though an increasing number of studies revealed acupuncture's therapeutic effects both in clinical and animal experiments research for PD (12–15).

Bibliometric analysis is performed with visualization tools to analyze the numerous published academic literature, which has advantages over other conventional research methods, such as study review, meta-analysis, and experimental or clinical research, which are not able to reach the same depth analysis results (16–20). With the help of visualization applications, namely, CiteSpace and VOSviewer software, bibliometric analysis can qualitatively and quantitatively explore the contribution of authors, countries/regions, institutions, and their cooperation relationships. More importantly, bibliometric analysis can point out the hotspots and frontiers and also predict the development trends of a specific field, which could be an important indicator for the following research in the future (21, 22).

Studies have revealed the therapeutic effect of the treatment of acupuncture for PD over the past 20 years (23, 24). However, there is no research on the bibliometric analysis of the global development of acupuncture on PD. It is important to understand the global trend in the research and development of acupuncture treatment for PD. This study was designed to explore the development of acupuncture for PD worldwide based on the Web of Science Core Collection (WoSCC) database, including clinical research and animal experiments from 2000 to 2021. The global research trends and topic hotspots will be reflected by identifying the core authors and their research cooperation relationships, institutions, countries and regions, and network analysis of keywords.

## Methods

All the data were extracted from the Web of Science Core Collection database based on the requirements of CiteSpace and VOSviewer software. The Web of Science Core Collection database was selected for this study for its wide range of comprehensive literature and high citation rates. The following search strategy was used in this study to generate the search result: “TS = (Parkinson disease or Parkinson's disease or Parkinsonism or Parkinson^*^) and TS = [Acupuncture or Electroacupuncture (EA) or Acupuncture^*^],” with the publication time from 2000 to 2021. The document type was limited to articles and reviews, without limitation of the language.

Two authors (LXP and WW) retrieved the publications and assessed the articles independently. Any uncertainty about the eligibility of the article will be consulted by a third reviewer (WQ). The VOSviewer and CiteSpace were applied to conduct the analysis. The analysis of the annual publications, the top countries/regions, institutions, authors, journals, and keywords were performed in the results of the network visualization. The parameter of the VOSviewer was set as follows: Method (LinLog/modularity). The parameters of CiteSpace were set as follows: Method (LLR), time slicing (2000–2021), years per slice (1), term source (all selection), node type (choose one at a time), selection criteria (top 50 objects), and pruning (pathfinder).

## Results

### Analysis of Annual Publications

**Figure 1:**
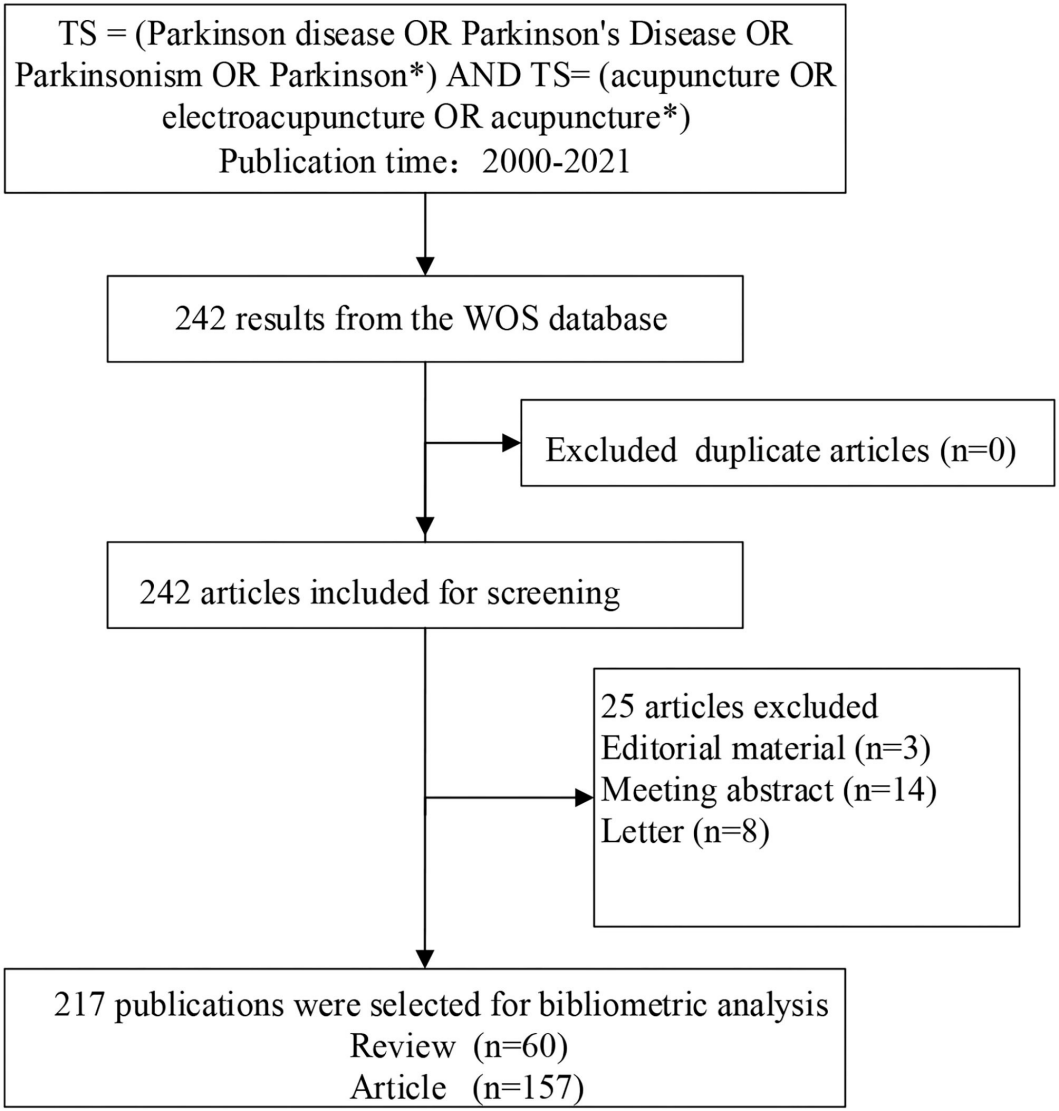
Flowchart of the selection process for the eligible literature.

**Figure 2:**
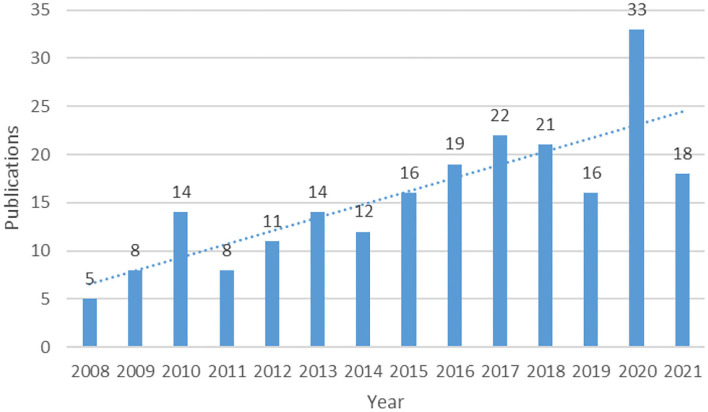
The number of annual publications on acupuncture treatment for Parkinson's disease (PD) from 2000 to 2021. The horizontal coordinate represents the different years and the vertical coordinate represents the single-year publication number.

There are 217 publications (157 articles and 60 reviews) that were collected from the WoSCC database in this study (Figure 1). The output of the publication grew slowly and showed fluctuations from 2008 to 2014. After 2015, the annual publication showed an upward tendency with more than 15 papers published each year. Notably, the output of 2020 was more than twice that of 2019 and reached its peak with 33 articles (Figure 2).

### Analysis of Countries/Regions

**Figure 3:**
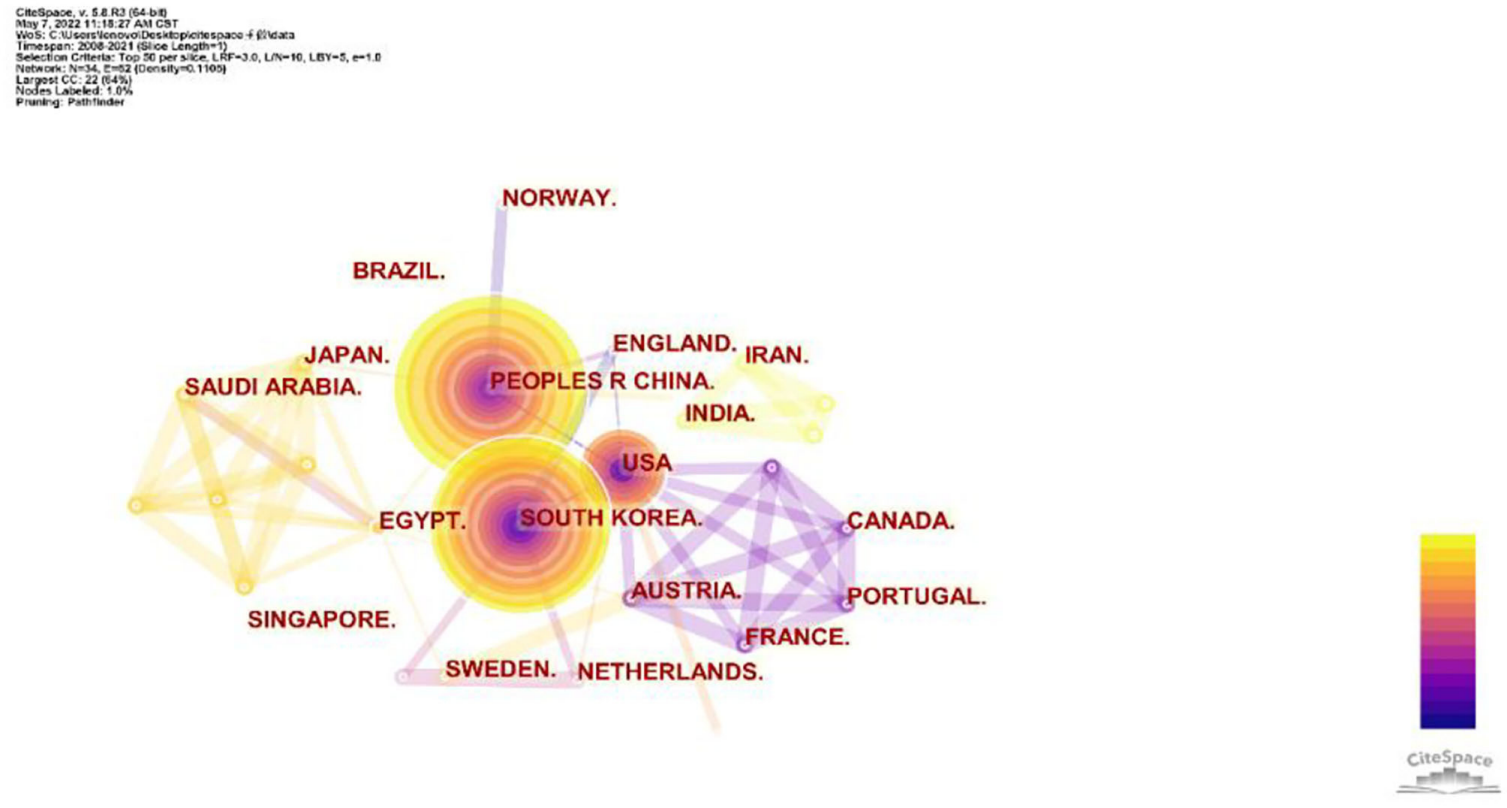
Network map of countries/regions from Citespace. Each node represents a country/region and the size of the node presents the number of publications of that country/region. Connections between nodes present the collaborations between the regions/countries and the thickness of the lines indicates the strength of the relationship. The purple ring in the inner circle represents the publications from the earliest year and the yellow ring in the outer circle is the most recent publications.

A total of 34 countries/regions were involved in the publications of PD treated by acupuncture. China (101, 46.5%), South Korea (68, 31.3%), the United States (39, 17.9%), England (8, 3.7%), and Egypt (7, 3.2%) were the top five countries/regions in terms of the number of publications (Figure 3). The documents from China were almost half of the total. The countries/regions with the top three total link strengths were China, the United States, and South Korea, which mean that they had more cooperation with other countries (Table 1). Those three countries also had the largest number of citations when ranked by citations. Those three leading countries/regions made great contributions to this field.

**Table 1:** Top 5 countries/regions for publications of acupuncture for Parkinson's disease (PD).

**Rank**	**Countries/Regions**	**Publications**	**Citations**	**Total link strength**
1	China	101	1,314	31
2	South Korea	68	1,255	15
3	USA	39	1,260	29
4	England	8	291	5
5	Egypt	7	136	11

### Analysis of Institutions

**Figure 4:**
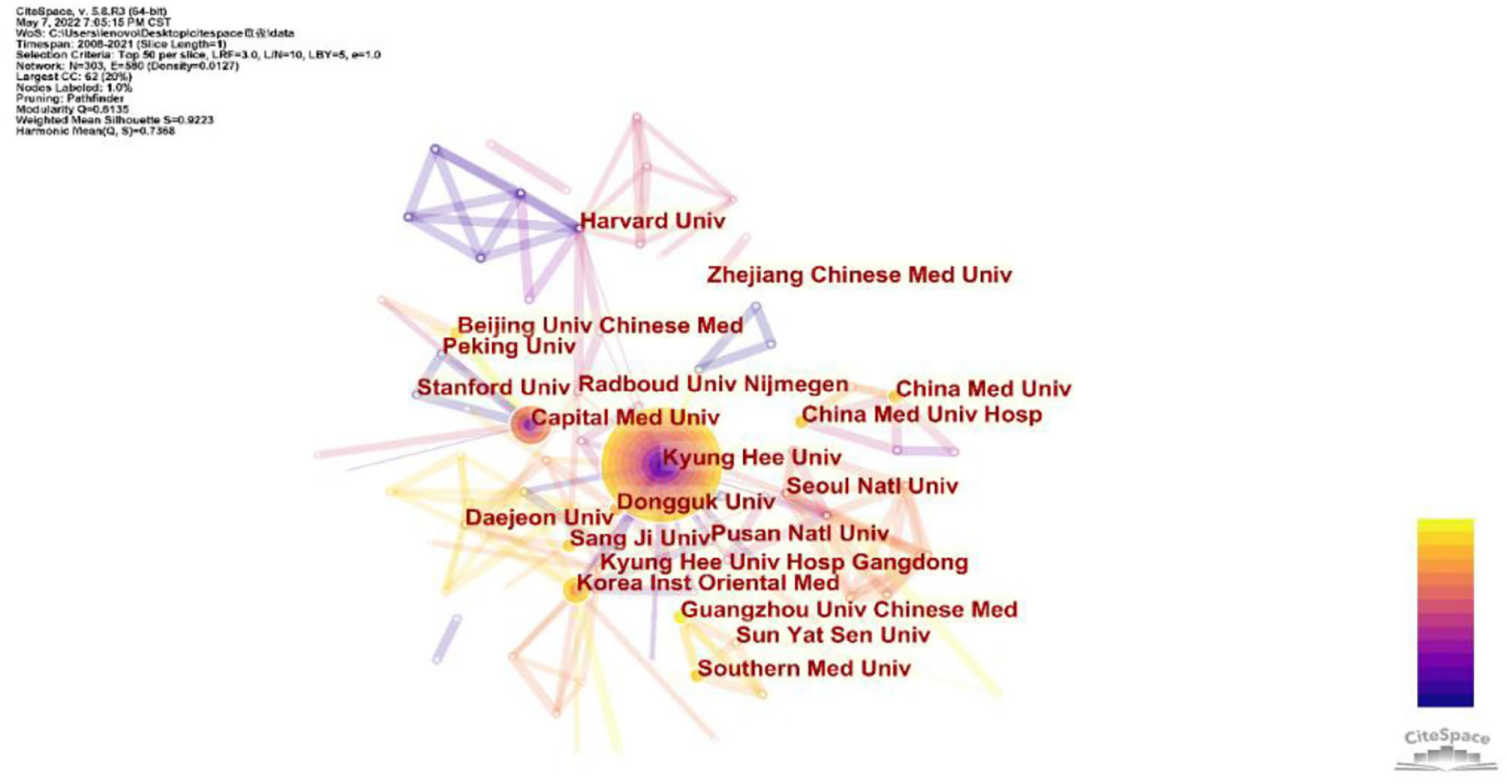
Network map of institutions from Citespace. Each node represents an institution and the size of the node presents the number of publications of that institution. Connections between nodes present the collaborations between the institutions and the thickness of the lines indicates the strength of the cooperation. The purple ring in the inner circle represents the publications from the earliest year and the yellow ring in the outer circle is the most recent publications.

According to the results of the analysis, 303 institutions were concluded to research of acupuncture on PD (Figure 4). Kyung Hee University has the largest number of publications (49, 22.5%), followed by Capital Medical University (24, 11%), China Medical University (13, 5.9%), Korea Institute of Oriental Medicine (13, 5.9%), China Medical University Hospital (11, 5%), and Pusan National University (11, 5%). The top 10 institutions with the greatest number of cited times are given in Table 2. Kyung Hee University was the most cited institution (1,033), followed by Capital Medical University (454) and Pusan National University (240). Institutions with the leading three link strengths are Kyung Hee University, Capital Medical University, and China Medical University. These institutions had more cooperation than other institutions.

**Table 2:** Top 10 institutions for publications on acupuncture for PD.

**No**	**Institutions**	**Count**	**Citations**	**Total link strength**
1	Kyung Hee University	49	1033	69
2	Capital Medical University	24	454	36
3	China Medical University	13	116	31
4	Korea Institute of Oriental Medicine	13	145	30
5	China Medical University Hospital	11	89	20
6	Pusan National University	11	240	8
7	Guangdong university of Chinese medicine	7	30	17
8	Sang Ji University	7	30	10
9	Dongguk University	6	112	11
10	Sanford university	6	146	14

### Analysis of Authors and Co-cited Authors

A total of 1,000 authors with 217 publications were involved in the field. Park Hi-Joon from Kyung Hee University in South Korea published the largest number of articles, followed by Xiaomin Wang from Capital Medical University in China and Sabina Lim from Kyung Hee University in South Korea (Table 3). They were the leading contributors to the publications in this field. Park Hi-Joon (592 citations) was the most cited author, followed by Hyejung Lee (379 citations), Coelho Miguel (372 citations), and Fox Susan H (372 citations). Park Hi-Joon was the most productive author with the largest number of publications and citations, which suggested that the studies of Park Hi-Joon in this field have been recognized by scholars (25–27). Co-cited authors are those two or more authors that are cited together in one publication and they build a co-citation relationship. Seung-Nam Kim (77) was the most frequently co-cited author, followed by Liang XiBin (74). There is an obvious cooperation network between different scholars, for example, Park Hi-Joon, Jung Woo-Sang, and Seung-Yeon Cho.

**Table 3:** Top 10 authors for publications on acupuncture for PD.

**No**	**Author**	**Count**	**Cited author**	**Citations**	**Co-cited author**	**Citations**	**Author**	**Total link strength**
1	Park, Hi-Joon	19	Hi-Joon Park	592	Seung-Nam Kim	77	Park Hi-Joon	60
2	Wang XiaoMin	18	Hyejung Lee	379	Liang, XiBin	74	Jung Woo-Sang	50
3	Sabina Lim	15	Coelho Miguel	372	Yeo, Sujung	68	Seung-Yeon Cho	50
4	Jia Jun	15	Fox, Susan H.	372	Park, Hi-Joon	61	Ko Chang-Nam	50
5	Yeo, Sujung	13	Goetz, Christopher G.	372	Cho, Seung-Yeon	60	Moon Sang-Kwan	50
6	Hyejung Lee	10	Katzenschlager, Regina	372	Jia, Jun	52	Lim Sabina	37
7	Seung-Nam Kim	9	Lim, Shen-Yang	372	Choi, Yeong-Gon	50	Park, Seong-uk	35
8	Chae, Younbyoung	8	Poewe, Werner	372	Chae, Younbyoung	46	Yeo Sujung	34
9	Chang-Nam Ko	8	Rascol, Olivier	372	Wang, Haomin	45	Cho, Ki-ho	32
10	Cho, Seung-Yeon	8	Ravina, Bernard	372	Jeon, Songhee	43	Kim Seung-nam	32

### Analysis of Journals and Co-cited Journals

A total of 116 academic journals were involved in the studies of acupuncture for PD. *Evidence-based Complementary and Alternative Medicine* contributed the largest number of publications (18, 8.2%) and *Movement Disorders* had the highest impact factor among the top 10 journals (IF = 10.338) (Table 4). Co-citation analysis of journals is to identify the journals with notable influence in a domain. *Movement Disorders* had the highest level of co-citations (529), followed by *Neuroscience Letters* (249), which demonstrated that *Movement Disorders* had an important position in this field. According to the Journal Citation Report (JCR) 2021 standards, three of the top 10 journals were categorized as the first quartile (Q1), three of the top 10 journals belonged to the second quartile (Q2), and four of the top 10 journals belonged to the second quartile (Q3). The top 10 journals were the mainstream journals in this field.

**Table 4:** Top 10 journals for publications on acupuncture for PD.

**No**	**Journal**	**Count**	**IF (2020)**	**JCR**	**Journals**	**Citations**	**IF (2020)**	**JCR**
1	Evidence Based Complementary and Alternative Medicine	18	2.63	Q2	Movement Disorders	529	10.338	Q1
2	Plos One	8	3.24	Q2	Neuroscience letters	249	3.046	Q3
3	Frontiers In Aging Neuroscience	8	5.75	Q1	Plos One	244	3.24	Q2
4	Acupuncture In Medicine	7	2.267	Q3	Brain Research	237	3.252	Q3
5	Medicine	7	1.889	Q3	Evidence Based Complementary and Alternative Medicine	205	2.63	Q2
6	Chinese Journal of Integrative Medicine	6	1.978	Q3	Frontiers In Neurology	192	4.003	Q2
7	Movement Disorders	5	10.338	Q1	Journal of Neuroscience Methods	178 2.39	Q3
8	Brain Research	4	3.252	Q3	Journal of Alternative and Complementary Medicine	170	2.582	Q2
9	CNS Neuroscience Therapeutics	4	5.243	Q2	Parkinsonism& Related Disorders	167	4.891	Q1
10	Parkinsonism and Related Disorders	4	4.891	Q1	Journal of Neuropathology and Experimental Neurology	159	3.685	Q2

### Analysis of Keywords

**Figure 5:**
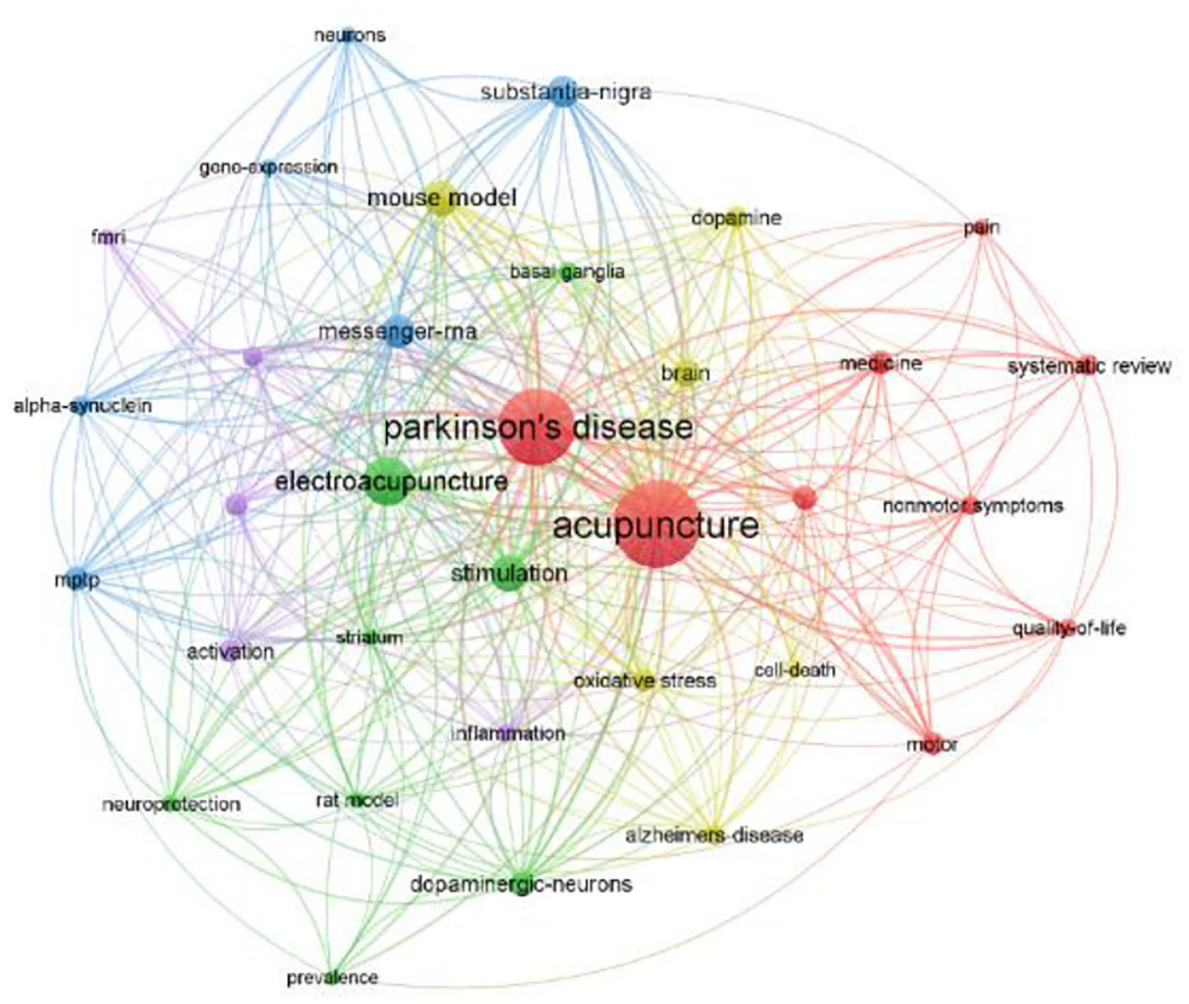
Network map of clustering keywords from VOSviewer. Each node represents a keyword and the size of the nodes represents the frequency of occurrences. The color of the nodes distinguishes the cluster of the keyword.

**Figure 6:**
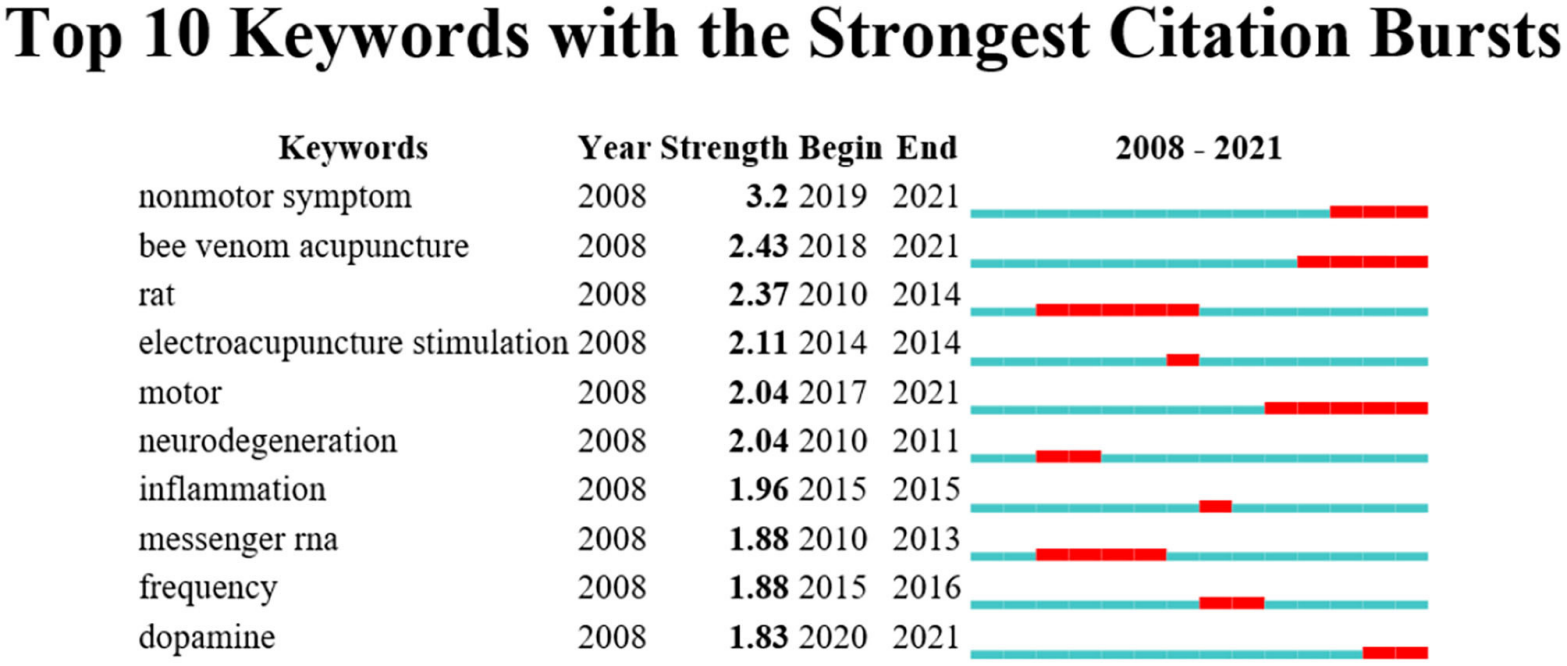
Top 10 keywords with the strongest citation bursts by CiteSpace. The beginning of a blue line represents when an article is published. The beginning of a red mark is the beginning of a period of burst and the end of the red mark is the end of the burst period.

The result of the keyword analysis helps to find research hotspots and predicts developing trends in a field. The top five most frequent keywords are acupuncture (35), Parkinson's disease (28), stimulation (21), electroacupuncture (20), and mouse model (20) (Table 5). As shown in the network map (Figure 5), the identified keywords can be divided into five clusters: acupuncture and bee venom acupuncture (BVA) (in red), electroacupuncture and neuroprotection (in green), alpha-synuclein and gene expression (in blue), cell death and mouse model (in yellow), and functional MRI (FMRI) and mechanism (in purple). Citation burst offers assistance to identify the hot words at the frontier of research within the entire analyzed period. The top five keywords with the strongest citation bursts were: Non-motor symptom (3.2), bee venom acupuncture (2.43), rat (2.37), electroacupuncture stimulation (2.11), and motor (2.04) (Figure 6).

**Table 5:** Top 10 keywords in the treatment of acupuncture for PD.

**Rank**	**Keywords**	**Count**
1	Acupuncture	35
2	Parkinson's disease	28
3	Stimulation	21
4	Electroacupuncture	20
5	Mouse model	20
6	Mechanism	14
7	Messenger RNA	14
8	Motor	13
9	Substantia nigra	13
10	Bee venom acupuncture	12

### Analysis of Citations

**Figure 7:**
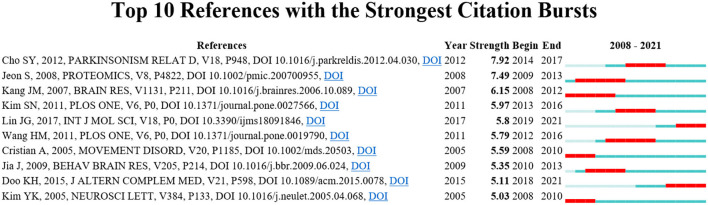
Top 10 references with the strongest citation bursts.

The co-citation analysis of references helps to identify the most frequently cited references and measures the relationships between references. Table 6 shows the top 10 most frequently co-cited references. The article published by Hi-Joon Park in *Experimental Neurology* had the largest number of citations (52), followed by the article published by Jun Mo Kang in *Brain Research* (45) and the article published by Lisa M Shulman in *Movement Disorders* (44). These references had a great influence on the treatment of acupuncture for PD. Publications with strong citation bursts had great influence in a field. Figure 7 presents the top 10 references with the strongest citation bursts.

**Table 6:** Top references in the treatment of acupuncture for PD.

**No**	**Title**	**Author**	**Year**	**Journal**	**Citations**
1	Acupuncture prevents 6-hydroxydopamine-induced neuronal death in the nigrostriatal dopaminergic system in the rat Parkinson's disease model	Hi-Joon Park	2003	Experimental Neurology	52
2	Acupuncture inhibits microglial activation and inf lammatory events in the MPTP-induced mouse model	Jun Mo Kang	2007	Brain Research	45
3	Acupuncture Therapy for the Symptoms of Parkinson's Disease	Lisa M. Shulman	2002	movement disorders	44
4	Effectiveness of acupuncture and bee venom acupuncture in idiopathic Parkinson's disease	Seung-Yeon Cho	2012	Parkinsonism and Related Disorders	44
5	Proteomic analysis of the neuroprotective mechanisms of acupuncture treatment in a Parkinson's disease mouse model	Songhee Jeon	2008	Proteomics	43
6	Evaluation of Acupuncture in the Treatment of Parkinson's Disease: A Double-Blind Pilot Study	Adrian Cristian	2005	movement disorders	42
7	Long-term high-frequency electro-acupuncture stimulation prevents neuronal degeneration and up-regulates BDNF mRNA in the substantia nigra and ventral tegmental area following medial forebrain bundle axotomy	Xi-Bin Liang	2002	Molecular Brain Research	41
8	The use of alternative therapies by patients with Parkinson's disease	Pam R. Rajendran	2001	Neurology	44
9	Acupuncture Enhances the Synaptic Dopamine Availability to Improve Motor Function in a Mouse Model of Parkinson's Disease	Seung-Nam Kim	2011	Plos One	36
10	Parsing Brain Activity Associated with Acupuncture Treatment in Parkinson's Diseases	Younbyoung Chae	2009	Movement Disorders	33

## Discussion

The number of annual publications is an important indicator to identify the general information of the most influential countries/regions, institutions, authors, and journals in the bibliometric analysis. The frequency of publication gradually increased from 5 publications in 2008 to 33 in 2020. The overall number of publications about acupuncture on PD showed an upward trend during the past two decades indicating that research on acupuncture on PD has got great attention from researchers, clinicians, and patients.

According to the analysis of the countries and organizations, China, South Korea, and United States were the leading countries in terms of the number of publications and citations. The documents from China were almost half of the total. If the database that we searched was extended to Chinese-based databases, China would have more published articles since acupuncture originated in China. China is the largest contributor and world leader in the research of acupuncture for PD. Kyung Hee University (South Korea) and Capital Medical University (China) were the top two organizations with the maximum number of publications and citations. It suggested that organizations from South Korea have made great progress during the past two decades. As shown in Figures 3, 4, the international cooperation of academic research between the top three productive countries has not yet been formed. Most collaborations of the publications were from domestic organizations. Therefore, the international cooperation of organizations from different countries needs to be strengthened to enhance the global development of acupuncture.

As for the analysis of authors and co-cited authors, Park Hi-Joon, from the Acupuncture and Meridian Science Research Centre of Kyung Hee University in South Korea, is the leading contributor in this field with the largest number of publications, citations, and cooperation, which indicate the outstanding studies he and his team had devoted during the past 20 years. As shown in Table 3, authors from South Korea had more cooperation with the domestic authors; however, the international cooperation of authors was not ideal. Therefore, authors worldwide should remove the language barriers to improve global cooperation in this field.

From the perspective of journals and co-cited journals, studies on acupuncture for PD are more popular in the journal of *Evidence-based Complementary and Alternative Medicine*, which is focused on the therapy of Traditional Chinese medicine. *Movement Disorders* had the highest number of co-citations and the most cited references were from high-quality journals, suggesting that scholars need to expand their choices to publish the research findings in high-quality journals, which help to gain more attention about acupuncture for PD worldwide.

As for the co-citation analysis of references, the most frequently co-cited article was published by Hi-Joon Park in *Experimental Neurology* in 2003 (52), which reported the neuroprotective effects of acupuncture.

The most frequent keywords are Acupuncture (35), Parkinson's disease (28), Stimulation (21), Electroacupuncture (20), Mouse model (20), Mechanism (14), Messenger RNA (14), Motor (13), Substantia nigra (13), and Bee venom acupuncture (12). The identified keywords can be divided into five clusters. The top five keywords with the strongest citation bursts were: non-motor symptom (3.2), bee venom acupuncture (2.43), rat (2.37), electroacupuncture stimulation (2.11), and motor (2.04). Based on the co-occurrence and the cluster analysis and the citation bursts of the keyword, we determine the academic hotspots and frontiers in this field.

### Clinical Manifestations of Parkinson's Disease

Parkinson's disease (PD) is recognized as the second most frequent neurological disease after Alzheimer's disease (28). The neuropathology hallmark of PD is the loss of dopaminergic neurons in the substantia nigra pars compacta (SNpc) and the presence of Lewy bodies (LBs), which are mainly composed of α-synuclein (α-Syn) (5, 29). Most of the LBs aggregates in the SNpc, however, are also found in the autonomic and peripheral nervous systems, which may be associated with the occurrence of some non-motor symptoms. Parkinson's disease is clinically characterized by the movement disorders such as postural instability, resting tremor, bradykinesia, muscle weakness, and muscular rigidity (30). However, non-motor symptoms from multisystem, namely, the impairments of gastrointestinal (GI), respiratory, genitourinary, cardiovascular, neuropsychiatric, sensory, skin, visual, pain, and other systems, not only challenge the patient quality of life, but also deteriorate the motor symptoms of PD (31, 32). Different from the motor symptoms, which become apparent when 50–70% of dopaminergic neurons in the central nervous system (CNS) already degenerate, some non-motor symptoms have been reported to occur up to 10 years before the clinical diagnosis (33). In contrast to the motor symptoms of PD, for which meditation is available and effective in clinical studies, non-motor symptoms are always poorly recognized and non-responsive to anti-PD meditations treatment even undertreated. Most of the drugs for PD treatment always cause non-motor symptoms as adverse effects, especially in elderly patients. However, non-motor symptoms of PD started to get more and more attention from clinicians, patients, and researchers, which offer adequate treatments for patients with PD to improve their quality of life and extend life expectancy. Recent research has paid more attention to the non-motor symptoms compared to the previous research that focused more on the motor symptoms. However, the etiology of non-motor dysfunctions in PD has not been fully understood. To better understand the potential mechanism of the non-motor manifestations and explore effective treatments, more and more animal models are emerging to investigate the efficacy and safety of promising therapies. However, no ideal animal model has been agreed upon for PD research (34). It is difficult to establish an appropriate model that fully reflects the non-motor features of human PD.

### Mechanisms of Acupuncture for Parkinson's Disease

Increasing evidence showed that acupuncture has potential effects on patients with PD to alleviate their motor and non-motor symptoms (15, 35). We summarized the possible mechanisms of acupuncture about how to exert protective effects on dopaminergic neurons: (1) Acupuncture could inhibit the increase of α-syn and accelerate the clearance of α-syn to reduce the abnormal aggregation of α-syn. Yeo et al. found that acupuncture at GB34 and LR3 upregulated serum/glucocorticoid-regulated kinase 1 (SGK1) and inhibited an α-syn production (36). Tian et al. (37) observed that microtubule-associated protein 1 light chain 3 II (LC3II) and lysosomal-associated membrane protein 1 (LAMP1) were reduced and more than 50% of α-syn in the SNpc was cleared after the treatment of acupuncture 4 days, suggesting that acupuncture at GB34 enhances degradation of α-syn and the clearance of autophagosomes. (2) Acupuncture inhibits the apoptotic pathways, thus promoting the survival of dopaminergic neurons. MA and acupoint injection at ST36 have been proven to inhibit the apoptotic pathway by downregulating the apoptotic markers such as Bax, cytochrome C, and upregulating B-cell lymphoma-2 (Bcl-2) (38). (3) Acupuncture can reduce the oxidative stress response of PD to protect the dopaminergic neurons. Yu et al. (39) proved that acupuncture could increase the levels of intracellular antioxidants such as superoxide dismutase (SOD), glutathione (GSH), and glutathione peroxidase (GSH-Px) and decrease the levels of malondialdehyde (MDA) to improve the rotarod behavior of PD. (4) Acupuncture could improve the movement disorders of PD by inhibiting the activation of microglia cells and normalizing the neuroinflammatory factors (34, 40). (5) Acupuncture could recover the homeostasis of the transmission of dopaminergic neurons in the basal ganglia circuit and attenuate dopaminergic neuronal loss (41, 42).

### Electroacupuncture

Electroacupuncture got more and more attention recently (43–45). Especially, in the clinical treatment, the electrical stimulation for 20 min always achieves a better clinical effect than the manual stimulation that needles are twirled or lifted and thrusted by hands for a few seconds during the 20 min treatment time. However, there are very few clinical trials that take the EA and manual acupuncture (MA) for comparison and the publications of the meta-analysis always conclude the clinical trials of the acupuncture without distinguishing between the electrical and the manual stimulation models. The frequency, intensity, and duration of the electrical and manual stimulation are mostly based on the patient's condition and it is quite difficult to make the parameters conform to the same standard in clinical trials. However, the comparison between the EA and the MA is easier to operate in the experimental research because the parameters of the stimulation, namely, the frequency, intensity, and duration, are quantifiable in the animal models in the experimental studies. To identify that the electric current was the only reason for the different clinical effects between these two stimulations (46), more and more well-designed clinical trials should be developed and applied when comparing EA vs. MA.

### Bee Venom Acupuncture

Bee venom acupuncture is a new treatment of acupuncture that injects the diluted bee venom into the acupoint or applies the bee venom on the tips of acupuncture needles. Bee venom consists of multiple substances, especially proteins and peptides, which have the effects of anti-inflammatory, antioxidant, neuroprotective, and antitumor. BVA has been used to relieve a range of pain conditions such as lumbar disk disease, osteoarthritis of the knee, and rheumatoid diseases. BVA can also alleviate neurological conditions, namely, peripheral neuropathies, stroke, and Parkinson's disease (47, 48). The clinical trial of BVA for idiopathic Parkinson's disease conducted by Cho et al. (49) concluded that BVA significantly improved motor symptoms in patients with PD. BVA treatment also reported the neuroprotective effect by normalizing all the neuroinflammatory and apoptotic markers and restoring brain neurochemistry in rotenone-induced PD mice models (50). However, there are certain problems regarding its safety and the possible appearance of adverse effects. Therefore, practitioners of bee venom therapy should be cautious when applying it in daily clinical practice.

## Conclusion

This study unveiled the research status of acupuncture on PD during the past two decades. Publications in this field were on the rise over time. The authors from different countries/regions and organizations need to remove the language and academic barriers to enhance global cooperation and communications. Most articles were published in journals, which are focused on the treatment of Traditional Chinese medicine, indicating that scholars need to publish the research findings in high-quality journals to gain more attention worldwide. This study also helps scholars to identify the influential journals for publication. Some issues remain unsolved, such as the mechanism leading to the non-motor symptoms of PD, the establishment of appropriate models that fully reflects the non-motor features of human PD, and the efficacy and safety of promising therapies for patients with PD. Those questions need to be put on the agenda, which offers assistance for the scientists to explore the new directions in further studies.

## Data Availability Statement

The original contributions presented in the study are included in the article/supplementary materials, further inquiries can be directed to the corresponding author/s.

## Author Contributions

All authors listed have made a substantial, direct, and intellectual contribution to the work and approved it for publication.

## Funding

This study was financially supported by the National Natural Science Foundation of China (Grant No. 81873388).

## Conflict of Interest

The authors declare that the research was conducted in the absence of any commercial or financial relationships that could be construed as a potential conflict of interest.

## Publisher's Note

All claims expressed in this article are solely those of the authors and do not necessarily represent those of their affiliated organizations, or those of the publisher, the editors and the reviewers. Any product that may be evaluated in this article, or claim that may be made by its manufacturer, is not guaranteed or endorsed by the publisher.
